# Serum hepcidin is associated with retinopathy of prematurity and modulates oxidative stress and angiogenic responses in retinal microvascular endothelial cells

**DOI:** 10.3389/fped.2026.1821139

**Published:** 2026-06-08

**Authors:** Hui Yang, Huiyun Chen, Fang Cheng, Jingbo Jiang

**Affiliations:** 1 Department of Neonatology, Shenzhen Children's Hospital, Shenzhen, Guangdong, China; 2 School of Pharmaceutical Sciences (Shenzhen), Sun Yat-sen University, Shenzhen, Guangdong, China

**Keywords:** angiogenesis, hepcidin, hypoxia, oxidative stress, retinopathy of prematurity

## Abstract

**Introduction:**

Retinopathy of prematurity (ROP) is a vasoproliferative retinal disorder in preterm infants driven by impaired vascular development, hypoxia, oxidative stress, and pathological angiogenesis. Current treatments mainly target VEGF signaling but do not directly address upstream oxidative injury. Hepcidin, a key iron-homeostasis regulator, may modulate redox balance and angiogenic activation. However, whether hepcidin is reduced in ROP and whether supplementation mitigates hypoxia-induced oxidative stress and abnormal angiogenesis in retinal endothelial cells remain unclear.

**Objective:**

To investigate the effects of hepcidin on human retinal microvascular endothelial cells (hRMECs) under oxidative stress and assess its translational potential in ROP.

**Methods:**

Clinical data were collected from 35 preterm infants with gestational age <36 weeks, including 24 non-ROP controls and 11 ROP infants. Gestational age, birth weight, ROP stage, and serum hepcidin were analyzed. hRMECs were exposed to 1% O_2_ to establish a hypoxia model. Cell viability, VEGFA and HIF-1α mRNA expression, secreted VEGFA, intracellular reactive oxygen species (ROS), transcriptomic changes, and tube formation were evaluated. Hepcidin was added under hypoxia to assess its effects on oxidative stress and angiogenic activation.

**Results:**

Infants with ROP had lower gestational age and birth weight than controls, and gestational age differed across ROP stage groups. Serum hepcidin-related signals were numerically lower in ROP infants, but no significant stage-dependent decrease was observed. In hRMECs, 1% O_2_ altered cell viability, upregulated VEGFA and HIF-1α mRNA, increased secreted VEGFA, and promoted ROS accumulation, confirming a hypoxia-induced endothelial stress model. Transcriptomic analysis suggested that hepcidin affected hypoxia-responsive metabolic pathways, including cysteine and methionine, selenocompound, and tryptophan metabolism. Functional validation showed that hepcidin reduced VEGFA at transcript and secreted protein levels, decreased intracellular ROS, and suppressed hypoxia-induced tube formation by reducing junction points, branch points, and total tube length.

**Conclusion:**

This study provides preliminary clinical and experimental evidence supporting a potential role of hepcidin-related signaling in ROP. Although the clinical findings are exploratory, the *in vitro* results indicate that hepcidin can attenuate hypoxia-induced oxidative stress and pathological angiogenic activation in retinal endothelial cells. Hepcidin may represent a biologically relevant pathway and potential candidate for further biomarker and therapeutic research in ROP.

## Graphical Abstract

**Figure:**
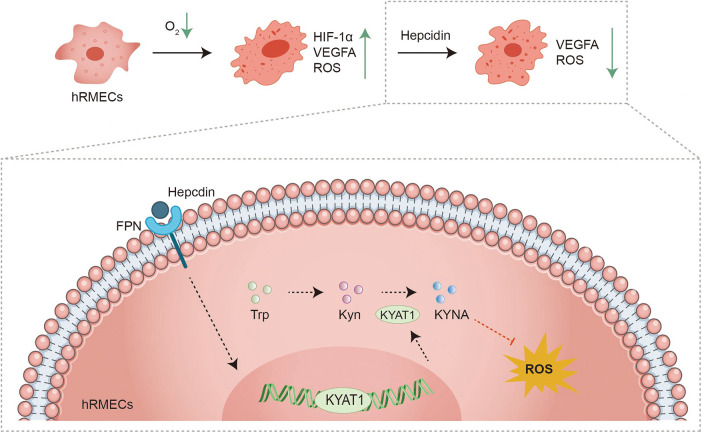
Proposed working model illustrating the potential mechanism by which hepcidin attenuates oxidative stress and angiogenic responses in hypoxic human retinal microvascular endothelial cells (hRMECs). Under hypoxic conditions, hRMECs exhibit increased oxidative stress and enhanced angiogenic activity. Hepcidin treatment attenuates these pathological changes. Based on the transcriptomic findings, we hypothesize that hepcidin may regulate KYAT1 expression in hRMECs and thereby influence the tryptophan metabolic pathway. In this proposed pathway, tryptophan (Trp) is converted to kynurenine (Kyn), which is further metabolized by KYAT1 to generate kynurenic acid (KYNA). KYNA may contribute to the reduction of intracellular reactive oxygen species (ROS), thereby suppressing hypoxia-induced oxidative stress and angiogenic activation. The upper panel summarizes the observed phenotypic changes, whereas the enlarged lower panel illustrates the proposed intracellular pathway. Dashed arrows indicate hypothetical mechanistic links that have not been directly validated in the present study.

## 1 Introduction

Retinopathy of Prematurity (ROP) is a leading cause of blindness in premature infants, primarily driven by immature retinal vascular development and hypoxia-induced pathological angiogenesis (1). With advancements in neonatal intensive care, the survival rate of premature infants has significantly increased; however, this has also led to a rising incidence of ROP, making it one of the foremost causes of childhood visual impairment and blindness worldwide (2–4). The pathogenesis of ROP involves abnormal vascular constriction, obstruction, and subsequent pathological neovascularization, which can ultimately result in vitreous hemorrhage, retinal detachment, and even blindness (5, 6). The pathogenesis of ROP is complex, involving interactions among various cells and molecular pathways. Hypoxia serves as a critical trigger for ROP development. Under hypoxic conditions, retinal endothelial cells experience oxidative stress, inflammatory responses, and metabolic dysregulation, which ultimately lead to pathological angiogenesis (7, 8). Research has demonstrated that hypoxia-inducible factor-1α (HIF-1α) and vascular endothelial growth factor (VEGF) play pivotal roles in the pathological progression of ROP (8). While current treatments, such as laser photocoagulation and anti-VEGF therapy, have demonstrated efficacy, challenges remain, including high recurrence rates and potential adverse effects (9–11). Therefore, identifying novel therapeutic targets and elucidating underlying mechanisms are crucial for improving ROP management.

Hepcidin is a key hormone synthesized in the liver that regulates iron metabolism. Its primary function is to maintain systemic iron homeostasis by modulating iron absorption, release, and storage (12, 13). Recent studies have shown that hepcidin alleviates oxidative stress and inflammatory responses by regulating the expression of iron metabolism-related genes (14, 15), suggesting its potential therapeutic role in ROP. However, the specific mechanisms by which hepcidin acts in ROP remain incompletely understood.

In this study, we compared clinical data from infants with and without ROP and found that lower gestational age and birth weight were associated with ROP in this exploratory cohort. Serum hepcidin-related signals showed a numerically lower pattern in infants with ROP, but the clinical interpretation was limited by the assay quantification range. To explore the potential biological relevance of hepcidin in ROP, we established a hypoxia-induced human retinal microvascular endothelial cell (hRMECs) model. RNA sequencing combined with experimental validation showed that hepcidin attenuated hypoxia-induced retinal endothelial cell injury, at least in part, through modulation of antioxidant and anti-angiogenic pathways. Collectively, these findings suggest that hepcidin may serve as a potential biomarker and therapeutic target in ROP, and provide a basis for further translational and *in vivo* studies.

## 2 Materials and methods

### 2.1 Measurement of serum hepcidin levels by ELISA

Serum hepcidin concentrations were measured using a Human Hepc (Hepcidin) ELISA kit (Elabscience, Cat. No. E-EL-H6013, USA) according to the manufacturer's instructions. This sandwich ELISA assay has a sensitivity of 37.5 pg/mL and a detection range of 62.5–4,000 pg/mL. Serum samples were prepared by centrifugation at 1,000 × g for 10 min, and the supernatants were collected for analysis. Briefly, 100 μL of standards or serum samples was added to each well and incubated at 37° C for 90 min. After removal of the liquid, 100 μL of biotinylated detection antibody working solution was added to each well and incubated at 37° C for 1 h. The plate was then washed three times, followed by incubation with 100 μL of HRP conjugate working solution at 37° C for 30 min. After five washes, 90 μL of TMB substrate solution was added to each well and incubated at 37° C for 15 min in the dark. The reaction was stopped by adding 50 μL of stop solution to each well, and the optical density was measured immediately at 450 nm using a microplate reader. A standard curve was generated using serially diluted standards (4,000, 2,000, 1,000, 500, 250, 125, 62.5, and 0 pg/mL), and serum hepcidin concentrations were calculated accordingly. Each sample was assayed in triplicate, and the mean value was used for statistical analysis.

### 2.2 RNA sequencing

Total RNA was isolated from cells using TRIzol® Reagent according to the manufacturer's instructions. RNA integrity was assessed with a 5,300 BioAnalyzer (Agilent) and quantified using the ND-2000 (NanoDrop Technologies). Libraries were sequenced on an Illumina NovaSeq X Plus platform (Illumina, USA), and differential expression was analyzed with DESeq2. Sequencing data are available in the NCBI BioProject database under accession number PRJNA1238557.

### 2.3 Cell culture

hRMECs (Tongpai, Cat. No. hRMECs, China) were cultured in DMEM/F-12 medium (Gibco, Cat. No. 6123189, USA) supplemented with 10% fetal bovine serum (ExCell Bio, Cat. No. FSP500, China) and 1% penicillin-streptomycin. Cells were maintained at 37° C in a humidified incubator containing 21% O_2_ and 5% CO_2_, and cells between passages 15 and 18 were used for all experiments. The cells were used according to the supplier-provided cell identity and quality-control information, and no additional in-house authentication was performed.

### 2.4 Induced hypoxia model

To establish the *in vitro* hypoxia model, hRMECs were seeded and cultured overnight under normoxic conditions (21% O_2_, 5% CO_2_, 37° C) to allow stable attachment. The following day, cells were transferred to a tri-gas incubator (Heal Force, HF100) and exposed to hypoxic conditions (1% O_2_, 5% CO_2_, and 94% N_2_) at 37° C for 24 or 48 h. Cells maintained under normoxic conditions served as the control group. At the end of the indicated exposure period, cells were harvested for subsequent analyses to verify successful hypoxia induction.

### 2.5 Hepcidin treatment protocol

For hepcidin intervention experiments, hRMECs were first seeded and cultured overnight under normoxic conditions (21% O_2_, 5% CO_2_, 37° C) to allow stable attachment. The following day, cells were divided into three groups: the normoxia control group (NC), the hypoxia model group (PC), and the hypoxia plus hepcidin treatment group (HEPC). The NC group was maintained under normoxic conditions (21% O_2_, 5% CO_2_, 37° C) and treated with an equal volume of phosphate-buffered saline (PBS) for 24 h. The PC group was cultured under hypoxic conditions in a tri-gas incubator (1% O_2_, 5% CO_2_, and balanced N_2_, 94%) at 37° C and treated with an equal volume of PBS for 24 h. The HEPC group was exposed to the same hypoxic conditions and treated with hepcidin (GLPBIO, Cat. No. GA22331, USA) at a final concentration of 0.1 μg/mL for 24 h. After treatment, cells from each group were harvested for subsequent experiments.

### 2.6 RNA isolation and quantitative real-time PCR

Cellular RNA was extracted with TRIzol (Takara, Japan) and measured on a NanoDrop One (Thermo Fisher). cDNA was generated using EasyScript cDNA Synthesis SuperMix (TransGen, China), and qPCR was carried out with PerfectStart Green qPCR Mix (TransGen, China) on a Roche LightCycler 96. Expression was normalized to ACTIN and reported as fold change via the 2^−ΔΔCt^ method. Primer sequences are provided below. ACTIN: GCCGTCTTCCCCTCCA (forward, 5′-3′), CTCGTCGCCCACATAGGAA (reverse, 5′-3′); VEGFA: ATCTTCAAGCCATCCTGTGTGC (forward, 5′-3′), CAAGGCCCACAGGGATTTTC (reverse, 5′-3′); HIF-1α: CAGTCGACACAGCCTGGATA (forward, 5′-3′), TTCTTCTGGCTCATATCCCATCAA (reverse, 5′-3′).

### 2.7 Measurement of secreted VEGFA protein by ELISA

Secreted VEGFA protein levels in hRMEC culture supernatants were measured using a STARTER OneStep ELISA Kit for Human VEGF/VEGFA (STARTER, Cat. No. SOC3124, China) according to the manufacturer's instructions. After the indicated treatments, culture supernatants were collected and centrifuged at 2,000 × g for 10 min to remove cell debris. Standards were prepared with Assay Diluent #D1 to generate a standard curve ranging from 78.13 to 5,000 pg/mL, with 0 pg/mL as the blank control. The reported sensitivity of the assay was 17.123 pg/mL. Absorbance was measured at 450 nm using a microplate reader (BioTek, Epoch2), and VEGFA concentrations were calculated using a four-parameter logistic standard curve. VEGFA protein levels were normalized to the corresponding NC group and expressed as fold change. Each experiment was performed independently three times.

### 2.8 ROS level detection

Intracellular reactive oxygen species (ROS) levels were measured using the DCFH-DA fluorescent probe, followed by analysis with flow cytometry or fluorescence microscopy. After the indicated treatments, the culture medium was removed and the cells were gently washed with PBS. The cells were then incubated with 5 μM DCFH-DA (Sigma-Aldrich, Germany) working solution prepared in serum-free medium at 37° C for 30 min in the dark to ensure sufficient probe loading. After incubation, the cells were washed thoroughly with PBS to remove excess extracellular probe. For adherent cells, cells were collected by trypsinization and resuspended in PBS to prepare single-cell suspensions. Intracellular ROS levels were subsequently detected by flow cytometry (CytoFLEX, Beckman Coulter) or fluorescence microscopy (Nikon Eclipse Ti2-U) and subjected to further analysis.

### 2.9 Cell viability assay

Cell viability was assessed using a Cell Counting Kit-8 (CCK-8; APExBIO, USA) assay. Cells in the logarithmic growth phase were dissociated into single-cell suspensions and seeded into 96-well plates at a density of 3 × 10^3^ cells per well, with five replicate wells for each group. After allowing the cells to attach and recover, the indicated treatments were applied. At the end of treatment, the culture medium was removed, and the cells were gently washed with PBS. To minimize evaporation-related effects, 100 μL PBS was added to the peripheral wells, whereas 100 μL serum-free medium containing 10% CCK-8 reagent was added to the remaining wells. The plates were then incubated at 37° C under 21% O_2_ and 5% CO_2_ for 30 min. Absorbance was measured at 450 nm using a microplate reader (BioTek, Epoch2). Blank wells were included to correct for background absorbance from the medium and reagent, and relative cell viability was calculated based on the OD values.

### 2.10 Angiogenesis assay

Phenol red-free Matrigel (Corning, Cat. No. 356237, Lot No. 3039003, protein concentration 7.9 mg/mL, Corning, USA) was thawed overnight at 4° C. Before use, the Matrigel was mixed 1:1 with culture medium and added to 24-well plates at 50 μL per well, followed by incubation at 37° C for 30 min to allow gelation. hRMECs were then seeded onto the Matrigel-coated wells at a density of 1 × 10^5^ cells per well and cultured at 37° C for 4 h. Tube-like structures were imaged using a microscope (Nikon Eclipse Ti2-U) and analyzed with ImageJ software. Tube-like structures were analyzed using ImageJ software with the Angiogenesis Analyzer plug-in.

### 2.11 Statistical analysis

Normality of clinical quantitative variables was assessed using the Shapiro–Wilk test. Gestational age and birth weight were compared between the ROP and non-ROP groups using the independent-samples *t*-test. Stage-stratified comparisons among the non-ROP, ROP stage 1, ROP stage 2, and ROP stage 3 groups were performed using one-way analysis of variance (ANOVA). Categorical variables, including sex and hepcidin threshold categories, were compared using two-sided Fisher's exact test. For serum hepcidin analysis, 62.5 pg/mL was used as the lower limit of quantification (LLOQ), and 37.5 pg/mL was used as the limit of detection (LOD). Hepcidin values below these thresholds were analyzed as categorical variables. Raw ELISA-derived hepcidin signals were retained only for exploratory descriptive analysis. Spearman rank correlation analysis was used to evaluate the associations of gestational age, birth weight, and raw ELISA-derived hepcidin signal with ROP status and ordinal ROP stage. ROP status was coded as 0 for non-ROP and 1 for ROP. Ordinal ROP stage was coded as 0 for non-ROP, 1 for ROP stage 1, 2 for ROP stage 2, and 3 for ROP stage 3. Clinical data were analyzed using SPSS version 22.0.

All *in vitro* experiments were performed independently at least three times. Experimental data are presented as mean ± SD and were analyzed using GraphPad Prism 8.0. For comparisons between two groups, unpaired two-tailed t-tests were used. For comparisons among multiple groups, one-way ANOVA followed by appropriate *post hoc* multiple-comparison tests was performed. RNA-sequencing differential expression analysis was performed using DESeq2. A two-sided *P* value <0.05 was considered statistically significant.

## 3 Results

### 3.1 Clinical characteristics and threshold-based serum hepcidin analysis in preterm infants

**Figure 1:**
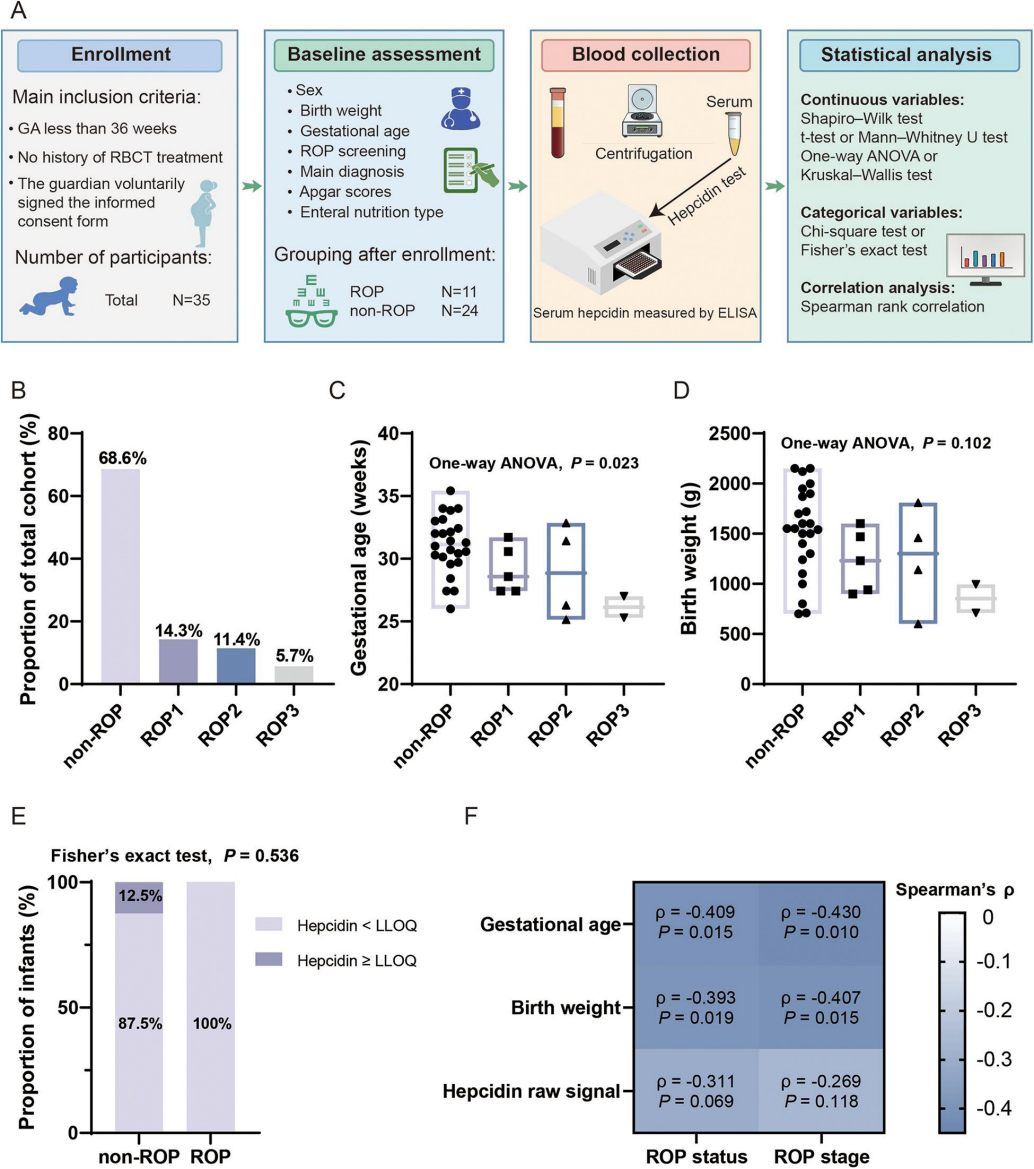
Clinical workflow and threshold-based serum hepcidin analysis in the preterm infant cohort. **(A)** Clinical sample data collection and testing process. **(B)** Distribution of non-ROP and ROP stage groups in the total cohort, *n* = 35. The cohort included 24 non-ROP infants, 5 infants with ROP stage 1, 4 with ROP stage 2, and 2 with ROP stage 3. **(C,D)** Stage-stratified distributions of gestational age and birth weight. Each dot represents one infant; boxplots show the median and interquartile range. Gestational age differed across groups, whereas birth weight showed a decreasing tendency without statistical significance. **(E)** Proportion of infants with serum hepcidin values below or at/above the lower limit of quantification (LLOQ), defined as 62.5 pg/mL. Fisher's exact test showed no significant difference between the non-ROP and ROP groups. **(F)** Spearman correlation heatmap showing associations of gestational age, birth weight, and hepcidin raw signal with ROP status and ROP stage. ROP status was coded as 0 = non-ROP and 1 = ROP; ROP stage was coded as 0 = non-ROP, 1 = ROP1, 2 = ROP2, and 3 = ROP3. Colors indicate Spearman's correlation coefficient *ρ*.

We enrolled 35 preterm infants with gestational age <36 weeks, including 24 non-ROP controls and 11 infants with ROP. Among the infants with ROP, 5 were classified as stage 1, 4 as stage 2, and 2 as stage 3 (Figures 1A,B). Categorized distributions of gestational age, birth weight, and sex are provided in Supplementary Table S1.

Compared with non-ROP infants, infants with ROP had lower gestational age (28.52 ± 2.71 vs. 31.10 ± 2.31 weeks, *P* = 0.006) and lower birth weight (1,168.64 ± 382.88 vs. 1,527.08 ± 435.29 g, *P* = 0.025), whereas sex distribution did not differ significantly between groups (*P* = 0.689; Table 1). In the stage-stratified analysis, gestational age differed across the non-ROP, ROP stage 1, ROP stage 2, and ROP stage 3 groups (one-way ANOVA, *P* = 0.023; Figure 1C). Birth weight showed a decreasing tendency across ROP stages but did not reach statistical significance (*P* = 0.102; Figure 1D). Detailed stage-stratified data are shown in Supplementary Table S2.

**Table 1:** Clinical characteristics and threshold-based serum hepcidin analysis in preterm infants with and without ROP.

Variable	ROP, *n* = 11	non-ROP, *n* = 24	*P* value
Gestational age, weeks	28.52 ± 2.71	31.10 ± 2.31	0.006
Birth weight, g	1,168.64 ± 382.88	1,527.08 ± 435.29	0.025
Sex, male/female	7/4	18/6	0.689
Hepcidin < LLOQ (<62.5 pg/mL)	11/11 (100.0%)	21/24 (87.5%)	0.536
Hepcidin < LOD (<37.5 pg/mL)	4/11 (36.4%)	3/24 (12.5%)	0.171

Data are presented as mean ± SD or *n*/*N* (%). Normality of gestational age and birth weight was assessed using the Shapiro–Wilk test, and both variables were compared between groups using the independent-samples *t*-test. Categorical variables, including sex and hepcidin threshold categories, were compared using two-sided Fisher's exact test. LLOQ was defined as 62.5 pg/mL, corresponding to the lower limit of the reported quantitative range of the ELISA assay. LOD was defined as 37.5 pg/mL, corresponding to the reported sensitivity threshold of the assay. Because most serum hepcidin measurements were below the LLOQ, hepcidin was primarily analyzed as categorical below-LLOQ data rather than as exact quantitative concentrations.

Because most serum hepcidin measurements were below the lower limit of quantification of the ELISA assay, hepcidin was primarily analyzed as categorical below-LLOQ data rather than as exact quantitative concentrations. LLOQ was defined as 62.5 pg/mL, and LOD was defined as 37.5 pg/mL. Hepcidin values below the LLOQ were observed in 21/24 non-ROP infants and 11/11 ROP infants, with no statistically significant difference between groups (Fisher's exact test, *P* = 0.536; Figure 1E; Table 1). Using the LOD threshold, 3/24 non-ROP infants and 4/11 ROP infants had hepcidin values below 37.5 pg/mL, with no statistically significant difference between groups (*P* = 0.171; Table 1). Although the primary below-LLOQ categorical analysis did not show a statistically significant difference between groups, raw ELISA-derived hepcidin signals were numerically lower in infants with ROP than in non-ROP controls (Supplementary Table S2; Supplementary Figure S1). Spearman correlation analysis showed that gestational age and birth weight were negatively correlated with both ROP status and ROP stage, whereas raw hepcidin signal showed only a non-significant negative trend (Figure 1F).

We further reviewed prematurity-related comorbidities and potential clinical confounders in both groups. No clearly documented primary systemic vascular disease, such as systemic vasculitis or hereditary systemic vascular malformation, was identified in the ROP cohort. Compared with the non-ROP group, the ROP group showed numerically higher proportions of several prematurity-related comorbidities, including bronchopulmonary dysplasia, intracranial or intraventricular hemorrhage, infection or inflammatory conditions, and NEC or severe gastrointestinal disease (Supplementary Table S3).

### 3.2 Establishment of a hypoxia model in hRMECs

**Figure 2:**
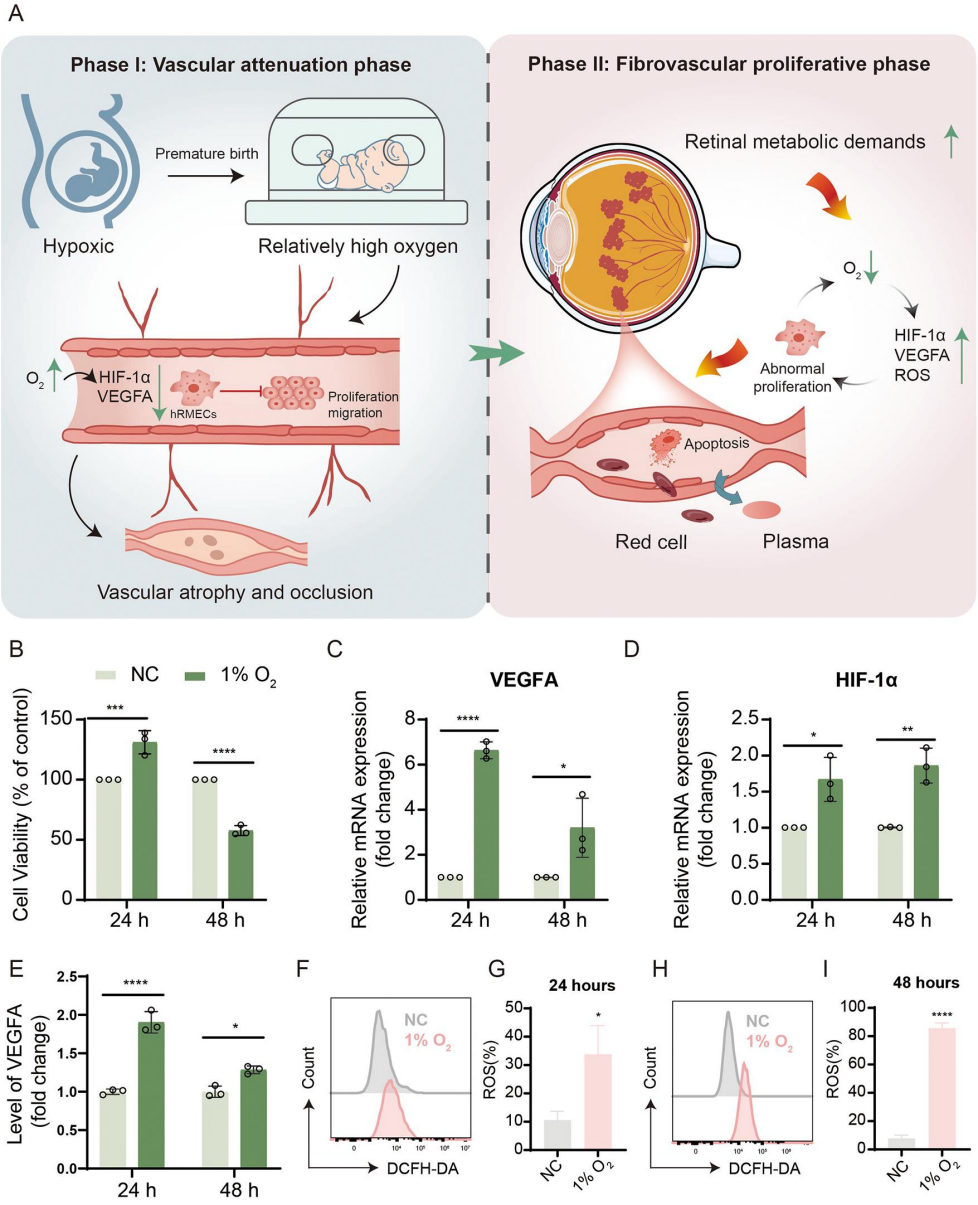
Pathogenesis of ROP and establishment of the hRMECs hypoxia model. **(A)** Schematic illustration of the two phases of ROP pathogenesis, including the vascular attenuation phase and the fibrovascular proliferative phase. **(B)** Cell viability of hRMECs after exposure to 1% O_2_ for 24 h and 48 h, as assessed by the CCK-8 assay (*n* = 3). **(C)** Relative mRNA expression of VEGFA in hRMECs after 24 h and 48 h of 1% O_2_ treatment compared with the corresponding normoxic control groups (*n* = 3). **(D)** Relative mRNA expression of HIF-1α in hRMECs after 24 h and 48 h of 1% O_2_ treatment compared with the corresponding normoxic control groups (*n* = 3). **(E)** Relative secreted VEGFA protein levels in hRMEC culture supernatants after 24 h and 48 h of 1% O_2_ treatment, as measured by ELISA and normalized to the corresponding normoxic control groups (*n* = 3). **(F,G)** Representative flow cytometry histogram and quantitative analysis of intracellular ROS levels after 24 h of 1% O_2_ treatment using the DCFH-DA probe (*n* = 3). **(H,I)** Representative flow cytometry histogram and quantitative analysis of intracellular ROS levels after 48 h of 1% O_2_ treatment using the DCFH-DA probe (*n* = 3). NC, normoxia control group; 1% O_2_, hypoxia treatment group. *P < 0.05, ** *P* < 0.01, *** *P* < 0.001, ****P < 0.0001.

The clinical course of retinopathy of prematurity (ROP) can be divided into two distinct phases (16). The first phase is characterized by vascular attenuation (Figure 2A, left panel). During this stage, preterm infants transition from the relatively hypoxic intrauterine environment to a comparatively hyperoxic extrauterine environment (17). This abrupt oxygen exposure suppresses the normal expression of HIF-1α and VEGFA. The reduction in these growth factors interrupts normal retinal vascular development, inhibiting the proliferation and migration of hRMECs. As a result, the vascular network in the peripheral retina becomes arrested, leading to vascular regression and occlusion and the formation of an avascular zone (18).

The second phase, fibrovascular proliferation (Figure 2A, right panel), ensues as the avascular retina continues to mature and its metabolic demands progressively increase (19). However, the occluded vessels cannot meet the oxygen requirements of this region, resulting in severe hypoxia. This pathological hypoxia strongly stimulates the overexpression of HIF-1α and VEGFA and promotes the generation of reactive oxygen species (ROS), thereby establishing a vicious cycle. Elevated VEGFA and other angiogenic factors induce abnormal neovascularization, producing fragile, disorganized vessels prone to plasma and erythrocyte leakage, and even apoptosis. These pathological neovessels, together with accompanying fibrotic tissue, form a fibrovascular membrane that may exert traction on the retina, ultimately leading to retinal detachment and blindness (18).

Since abnormal hRMEC responses under hypoxic conditions play a critical role in the pathogenesis of ROP, we established an *in vitro* hypoxia model by culturing hRMECs at 1% O_2_ (20). After 24 h of exposure to 1% O_2_, hRMECs showed significantly increased cell viability compared with the normoxic control group, suggesting a short-term hypoxia-induced adaptive response. In contrast, after 48 h of hypoxia, cell viability markedly decreased, indicating progressive hypoxia-induced cellular injury (Figure 2B).

We next examined hypoxia-responsive angiogenic signaling. The mRNA expression levels of VEGFA and HIF-1α were upregulated after both 24 h and 48 h of 1% O_2_ treatment compared with the corresponding normoxic control groups (Figures 2C,D). Consistently, ELISA analysis showed that secreted VEGFA protein levels in hRMEC culture supernatants were also increased under hypoxic conditions, further supporting activation of VEGFA-related angiogenic signaling in this hypoxia model (Figure 2E).

To further assess oxidative stress under hypoxia, we measured intracellular ROS levels in hRMECs using the DCFH-DA probe and flow cytometry. ROS levels were significantly elevated after 24 h and 48 h of 1% O_2_ treatment, with a more pronounced increase observed after 48 h of hypoxia compared with the normoxic control group (Figures 2F–I). Together, these findings indicate that 1% O_2_ exposure successfully induced hypoxia-related endothelial stress in hRMECs, characterized by altered cell viability, activation of HIF-1α/VEGFA signaling, increased secreted VEGFA protein, and ROS accumulation.

### 3.3 Hepcidin affects hRMECs under hypoxic conditions

**Figure 3:**
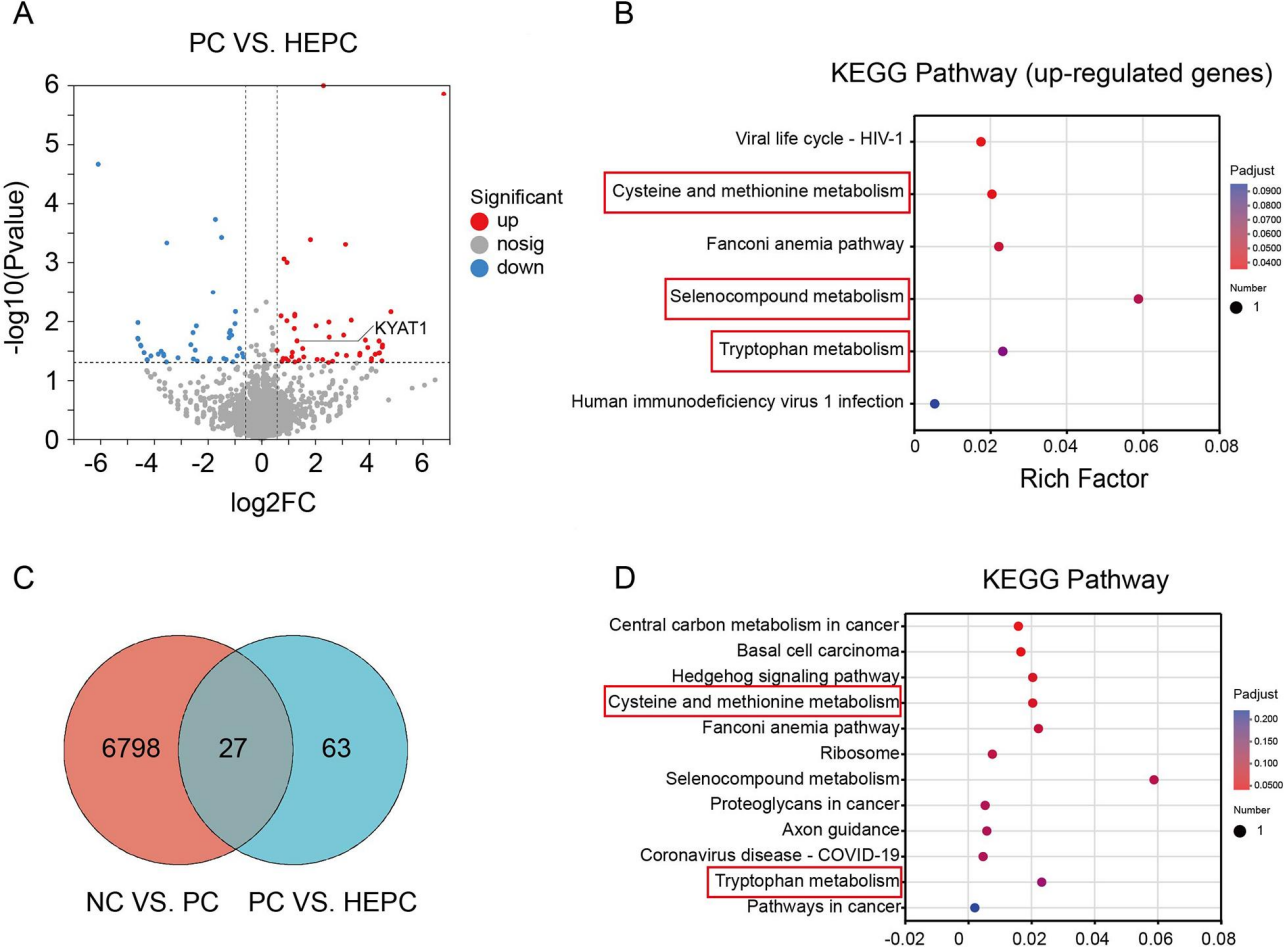
Hepcidin affects hRMECs under hypoxic conditions. **(A)** Volcano plot showing the differentially expressed genes between the hypoxia model group (PC) and the hypoxia plus hepcidin treatment group (HEPC) (*n* = 3). **(B)** KEGG enrichment analysis of up-regulated differentially expressed genes between the PC and the HEPC group (*n* = 3). **(C)** Venn diagram showing the differentially expressed gene sets between the control group (NC) and the PC group, as well as between the PC and the HEPC group (*n* = 3). **(D)** KEGG enrichment analysis of the common differentially expressed genes between NC and the PC group, as well as between the PC and the HEPC group (*n* = 3).

To investigate the potential role of hepcidin in ROP, we established a hypoxia model in hRMECs under 1% O_2_ for 24 h and performed RNA sequencing on cells from the normoxia control group (NC), the hypoxia model group (PC), and the hypoxia plus hepcidin treatment group (HEPC). Sequencing results revealed differential gene expression between the PC and HEPC groups (Figure 3A). Upregulated genes included TMLHE-AS1, ZNF843, KYAT1, GAGE12C, SERF1B, among others, whereas downregulated genes included RPL13AP5, CSPG4P10, PDF, SHC3, LINC00392, etc. (Table 2).

**Table 2:** Representative differentially expressed genes between the hypoxia model group (PC) and the hypoxia plus hepcidin treatment group (HEPC).

Gene	Regulation (HEPC vs. PC)
TMLHE-AS1	Upregulated
ZNF843	Upregulated
KYAT1	Upregulated
GAGE12C	Upregulated
SERF1B	Upregulated
RPL13AP5	Downregulated
CSPG4P10	Downregulated
PDF	Downregulated
SHC3	Downregulated
LINC00392	Downregulated

Further enrichment analysis of these differentially expressed genes indicated that KEGG pathway analysis of the upregulated genes was primarily enriched in cysteine and methionine metabolism, selenium compound metabolism, and tryptophan metabolism pathways (Figure 3B). By intersecting the differentially expressed genes between NC vs. PC and PC vs. HEPC groups (Figure 3C), and performing KEGG enrichment analysis on the overlapping genes, we observed consistent enrichment in cysteine and methionine metabolism and tryptophan metabolism pathways (Figure 3D), suggesting that hepcidin likely exerts its therapeutic effects through these two metabolic pathways.

### 3.4 Hepcidin exhibits antioxidant properties and regulates angiogenesis

**Figure 4:**
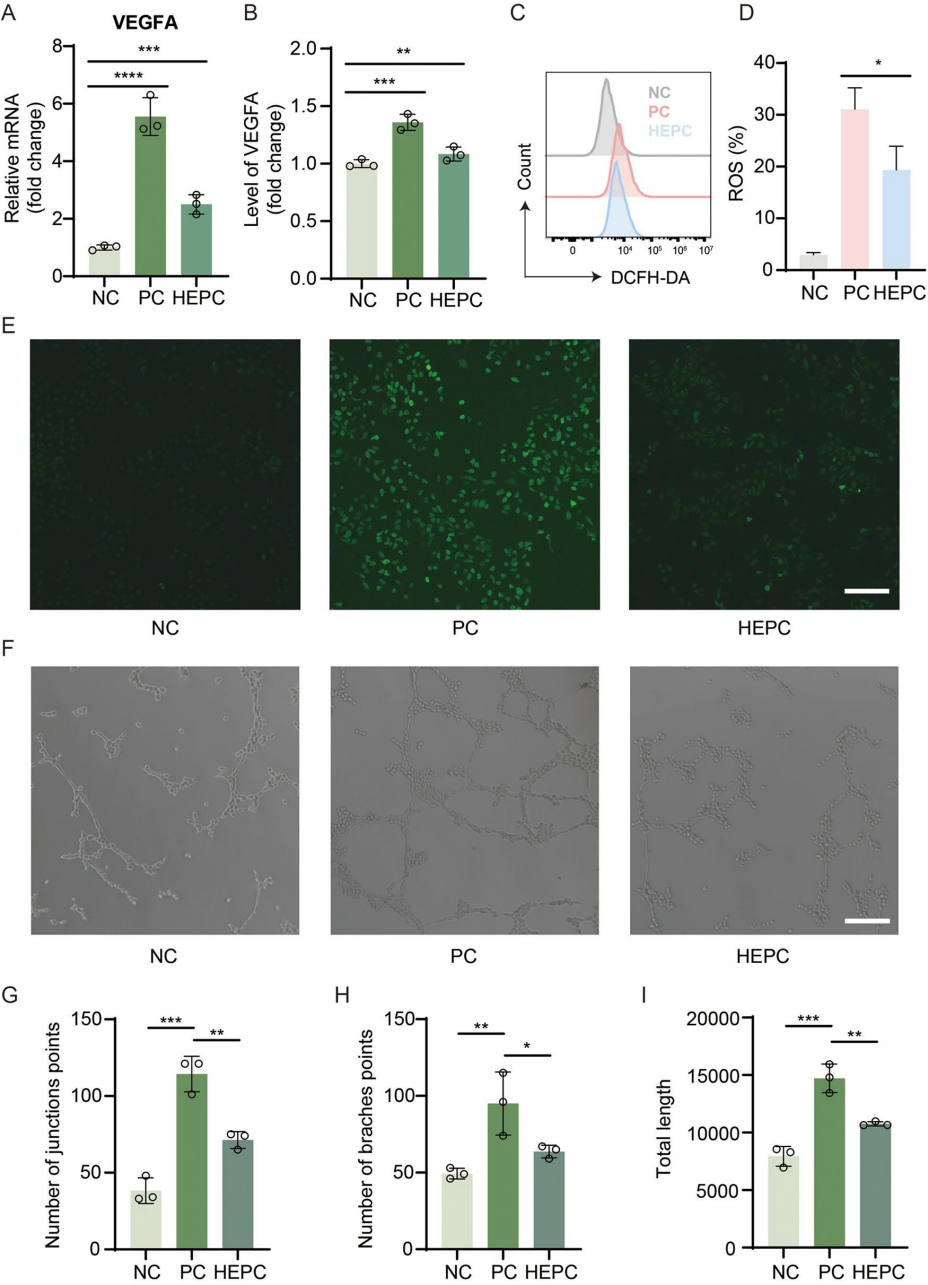
Hepcidin attenuates hypoxia-induced oxidative stress and angiogenic responses in hRMECs. **(A)** Relative mRNA expression of VEGFA in hRMECs under different treatment conditions (*n* = 3). **(B)** Relative secreted VEGFA protein levels in hRMECs culture supernatants, as measured by ELISA and normalized to the NC group (*n* = 3). **(C)** Representative flow cytometry histograms of DCFH-DA fluorescence under different treatment conditions (*n* = 3). **(D)** Quantitative analysis of intracellular ROS levels measured by flow cytometry (*n* = 3). **(E)** Representative fluorescence microscopy images of DCFH-DA staining in the NC, PC, and HEPC groups. Fluorescence intensity indicates intracellular ROS levels (*n* = 3). Scale bars = 500 μm. **(F)** Representative tube formation images of hRMECs in the NC, PC, and HEPC groups (*n* = 3). Scale bars = 500 μm. **(G–I)** Quantitative analysis of tube formation, including the number of junction points **(G)**, number of branch points **(H)**, and total tube length **(I)** (*n* = 3). NC, normoxia control group; PC, hypoxia model group; HEPC, hypoxia plus hepcidin treatment group. *P < 0.05, **P < 0.01, ***P < 0.001, ****P < 0.0001.

Building on the RNA sequencing results, we further validated the role of hepcidin through experimental assays. Under hypoxic conditions, VEGFA mRNA expression was markedly increased in hRMECs, whereas hepcidin treatment reduced VEGFA mRNA expression compared with the hypoxia model group (Figure 4A). Consistently, ELISA analysis showed that secreted VEGFA protein levels in hRMEC culture supernatants were also increased under hypoxia and were decreased after hepcidin treatment (Figure 4B), suggesting that hepcidin attenuates hypoxia-induced VEGFA activation at both transcript and protein levels.

To evaluate the antioxidant effect of hepcidin, we measured intracellular ROS using the DCFH-DA probe. Flow cytometry showed that ROS levels were increased in the hypoxia model group compared with the normoxia control group, while hepcidin treatment reduced ROS accumulation under hypoxic conditions (Figures 4C,D). Representative fluorescence microscopy images of DCFH-DA staining showed a similar pattern, with weaker fluorescence intensity in the hepcidin-treated group than in the hypoxia model group (Figure 4E).

We further assessed the effect of hepcidin on endothelial angiogenic activity using a tube formation assay. Hypoxia enhanced the tube-forming ability of hRMECs, whereas hepcidin treatment suppressed this hypoxia-induced angiogenic response (Figure 4F). Quantitative analysis confirmed that hepcidin significantly reduced the number of junction points, number of branch points, and total tube length compared with the hypoxia model group (Figures 4G–I). Together, these results indicate that hepcidin attenuates hypoxia-induced oxidative stress and abnormal angiogenic activation in hRMECs.

## 4 Discussion

ROP is characterized by disordered retinal vascular development in preterm infants. During the early phase of the disease, exposure to a relatively hyperoxic extrauterine environment suppresses pro-angiogenic signaling and interrupts normal vascular growth, whereas the later phase is driven by increasing retinal metabolic demand, relative hypoxia, oxidative stress, and pathological neovascularization (8). Because retinal endothelial cells are central effectors of these processes, hRMECs provide a relevant *in vitro* model for examining hypoxia-induced oxidative and angiogenic responses (8, 21).

In the present study, hypoxic exposure induced time-dependent changes in hRMEC behavior. Short-term hypoxia increased cell viability, whereas prolonged hypoxia reduced viability despite persistent upregulation of VEGFA and HIF-1α, suggesting that adaptive and injurious responses may coexist during sustained oxygen deprivation. This pattern may reflect progressive metabolic stress, excessive ROS accumulation, mitochondrial dysfunction, and activation of stress-related pathways such as p53 signaling (22). Consistent with this interpretation, hypoxia increased intracellular ROS levels and enhanced VEGFA-related angiogenic signaling, including both VEGFA mRNA expression and secreted VEGFA protein levels. These findings support the successful establishment of a hypoxia-induced retinal endothelial stress model that captures key features relevant to the proliferative phase of ROP.

The clinical analysis showed that lower gestational age and lower birth weight were more consistently associated with ROP than serum hepcidin-related measurements. Infants with ROP had significantly lower gestational age and birth weight than non-ROP infants, and Spearman correlation analysis further showed that both gestational age and birth weight were negatively correlated with ROP status and ordinal ROP stage. By contrast, raw ELISA-derived hepcidin signals showed only a non-significant negative trend with ROP status and were not significantly correlated with ROP stage. Moreover, the stage-stratified analysis did not show a monotonic decrease in hepcidin signal across increasing ROP stages. Therefore, the present clinical data do not support serum hepcidin as a validated marker of ROP severity.

The interpretation of serum hepcidin in this cohort requires caution. Most serum hepcidin measurements were below the lower limit of quantification of the ELISA assay; therefore, hepcidin was primarily analyzed as below-LLOQ categorical data rather than as exact quantitative concentrations. Although raw ELISA-derived hepcidin signals were numerically lower in infants with ROP, the below-LLOQ and below-LOD threshold analyses did not demonstrate statistically significant differences between ROP and non-ROP groups. Thus, serum hepcidin should be considered an exploratory biomarker candidate rather than a validated disease-specific marker in the current cohort. Future clinical studies should use more sensitive assays, such as ELISA kits with lower quantification limits or mass spectrometry-based methods, to more accurately define circulating hepcidin levels in preterm infants.

The causal relationship between altered hepcidin-related signals and ROP remains unresolved. Based on the clinical data alone, reduced hepcidin-related signals may reflect systemic prematurity-associated conditions rather than a direct cause of ROP. This interpretation is supported by the presence of multiple prematurity-related comorbidities and potential clinical confounders, including bronchopulmonary dysplasia, intracranial or intraventricular hemorrhage, anemia, iron supplementation, infection or inflammatory conditions, and NEC or severe gastrointestinal disease. No clearly documented primary systemic vascular disease was identified in the ROP cohort, but these comorbidities may influence systemic inflammation, oxygen exposure, erythropoietic activity, iron metabolism, and serum hepcidin interpretation. Therefore, the observed hepcidin-related clinical pattern may represent a consequence of prematurity-associated systemic alterations, ROP-related pathophysiology, or both.

Nevertheless, the *in vitro* findings provide biological plausibility for a functional role of hepcidin in retinal endothelial injury. Hepcidin treatment reduced hypoxia-induced VEGFA upregulation at both the transcript level and secreted protein level, decreased intracellular ROS accumulation, and suppressed endothelial tube formation. These results suggest that hepcidin can attenuate key pathological endothelial responses involved in oxidative stress and angiogenic activation. Therefore, although the clinical data cannot establish causality, the experimental data indicate that insufficient hepcidin-related signaling may create a permissive environment for hypoxia-driven oxidative and angiogenic injury in retinal endothelial cells.

RNA sequencing further suggested that hepcidin may influence hypoxia-responsive metabolic programs. Differentially expressed genes after hepcidin treatment were enriched in cysteine and methionine metabolism, selenocompound metabolism, and tryptophan metabolism. Among these pathways, tryptophan metabolism is of particular interest because KYAT1 is involved in the generation of kynurenic acid (KYNA), a metabolite reported to have antioxidant and anti-inflammatory properties (23). Based on these transcriptomic and functional data, we propose that hepcidin may modulate metabolic pathways related to redox homeostasis and endothelial activation. However, this model remains hypothesis-generating. Direct validation of pathway-specific metabolites, KYAT1 function, and downstream signaling will be required before a definitive mechanism can be established.

From a translational perspective, hepcidin may represent a potential modulator of hypoxia-induced oxidative stress and pathological angiogenesis in ROP. However, no clinical administration strategy can be proposed at this stage because the current evidence is limited to *in vitro* hRMEC experiments. Future translational studies should evaluate both local ocular delivery and systemic administration. Local ocular delivery, such as intravitreal injection, may provide greater retinal specificity and reduce systemic exposure, which is particularly important in fragile preterm infants. Conversely, systemic administration may be relevant because hepcidin is a circulating hormone and prematurity-associated iron, hypoxia, and inflammatory disturbances are systemic, but this approach may carry a higher risk of off-target effects on iron metabolism. Therefore, the optimal delivery route, effective dose range, pharmacodynamic profile, blood-retinal barrier penetration, and developmental safety must be carefully evaluated.

The use of serum rather than tear fluid for hepcidin measurement also requires consideration. Tear hepcidin or other ocular-fluid-based markers may offer greater ocular specificity. However, standardized tear collection from very preterm infants is technically difficult, often yields limited sample volume, and may cause discomfort or stress in a vulnerable neonatal population. Serum sampling was therefore selected because it was more clinically feasible and because hepcidin is primarily a systemic iron-regulatory hormone synthesized in the liver. Future studies comparing serum, tear, and other ocular compartments will be important to determine whether local ocular hepcidin better reflects retinal disease activity than systemic measurements.

Several limitations should be acknowledged. First, the clinical cohort was small, and the ROP stage subgroups were particularly limited, especially the stage 3 subgroup. This limited the statistical power of stage-stratified and correlation analyses. Second, serum hepcidin measurements were constrained by the quantification range of the ELISA assay, and most values were below the LLOQ. Third, only a single concentration of hepcidin, 0.1 μg/mL, was tested in this study. Based on the molecular weight of hepcidin, this dose corresponds to approximately 35.8 nM and was selected as a literature-informed working concentration according to previous evidence showing that the hepcidin–ferroportin axis is biologically active in the low nanomolar range. In primary hepatocytes, 350 nM synthetic hepcidin markedly reduced membrane-associated ferroportin after 24 h, and an effect was detectable at 10 nM (24). However, because no systematic dose-response analysis was performed in hRMECs, the present study does not establish 0.1 μg/mL as the optimal dose. Fourth, the clinical analysis was observational and based on single-time-point serum measurements; therefore, it cannot determine whether altered hepcidin-related signals precede ROP development or reflect systemic prematurity-related illness. Fifth, although VEGFA protein secretion was validated by ELISA, direct HIF-1α protein validation was not achieved, and conclusions regarding HIF-1α pathway activation rely on transcript-level and downstream VEGFA protein evidence. Sixth, although RNA sequencing suggested involvement of several metabolic pathways, pathway-specific metabolites and causal molecular mediators were not directly validated. Finally, the effects of hepcidin were evaluated only in an *in vitro* hRMEC hypoxia model and have not yet been confirmed in an *in vivo* ROP model.

In summary, the present study provides a combined clinical and experimental investigation of hepcidin-related signaling in ROP. Clinically, lower gestational age and birth weight were consistently associated with ROP, while serum hepcidin-related findings remained exploratory. Experimentally, hepcidin attenuated hypoxia-induced ROS accumulation, VEGFA activation, and abnormal angiogenic responses in hRMECs. These findings suggest that hepcidin-related signaling may be a biologically relevant pathway in ROP and support further investigation using larger longitudinal cohorts, more sensitive hepcidin assays, *in vivo* ROP models, dose-response studies, and targeted mechanistic validation.

## 5 Conclusion

In this study, we combined clinical observations with *in vitro* experimental models to investigate the potential role of hepcidin-related signaling in retinopathy of prematurity (ROP). Clinically, lower gestational age and birth weight were consistently associated with ROP, whereas serum hepcidin-related findings should be interpreted as exploratory. Raw ELISA-derived hepcidin signals showed a numerically lower pattern in infants with ROP, but these findings did not establish serum hepcidin as a validated disease-specific marker or severity indicator.

*In vitro*, hypoxia induced retinal endothelial stress characterized by altered cell viability, VEGFA activation, increased secreted VEGFA protein, ROS accumulation, and enhanced angiogenic activity. Hepcidin treatment attenuated these hypoxia-induced responses by reducing VEGFA expression at both transcript and secreted protein levels, decreasing intracellular ROS accumulation, and suppressing abnormal tube formation in hRMECs. Transcriptomic analysis further suggested that hepcidin may influence hypoxia-responsive metabolic pathways related to redox homeostasis and endothelial activation.

Taken together, these findings provide preliminary clinical and experimental evidence supporting hepcidin-related signaling as a biologically relevant pathway in ROP. Although the clinical findings remain exploratory and the functional data require *in vivo* validation, this study suggests that hepcidin may have potential value as a candidate pathway for future biomarker development and therapeutic research in ROP. Further studies using larger longitudinal cohorts, more sensitive hepcidin assays, *in vivo* ROP models, dose-response designs, and targeted mechanistic validation are warranted.

## Data Availability

The datasets presented in this study can be found in online repositories. The names of the repository/repositories and accession number(s) can be found in the article/Supplementary Material.
