# Supplementary material for: Serum hepcidin is associated with retinopathy of prematurity and modulates oxidative stress and angiogenic responses in retinal microvascular endothelial cells
Source: Front Pediatr. 2026 Jun 8;14:1821139. doi: 10.3389/fped.2026.1821139 (PMC13283981; doi:10.3389/fped.2026.1821139)
Supplement: Supplementary file 1 [file Table1.docx]

**Supplementary Table S1. Categorized distributions of gestational age, birth weight, and sex in infants with and without ROP**

|  |  | ROP, *n* = 11 | non-ROP, *n* = 24 |
| --- | --- | --- | --- |
| Gestational age |  |  |  |
|  | < 28w | 6/11 (54.5%) | 3/24 (12.5%) |
|  | 28w - < 32w | 4/11 (36.4%) | 11/24 (45.8%) |
|  | 32w - < 36w | 1/11 (9.1%) | 10/24 (41.7%) |
| Birth weight |  |  |  |
|  | < 1000g | 5/11 (45.5%) | 3/24 (12.5%) |
|  | 1000g - < 1500g | 4/11 (36.4%) | 5/24 (20.8%) |
|  | 1500g - < 2000g | 2/11 (18.2%) | 16/24 (66.7%) |
| Sex |  |  |  |
|  | Male | 7/11 (63.6%) | 18/24 (75.0%) |
|  | Female | 4/11 (36.4%) | 6/24 (25.0%) |

Table note: Data are presented as n/N (%). This table provides categorized distributions of gestational age, birth weight, and sex for descriptive purposes. Continuous comparisons of gestational age and birth weight between ROP and non-ROP groups are reported in the main text and Table 1.
