# Supplementary material for: Serum hepcidin is associated with retinopathy of prematurity and modulates oxidative stress and angiogenic responses in retinal microvascular endothelial cells
Source: Front Pediatr. 2026 Jun 8;14:1821139. doi: 10.3389/fped.2026.1821139 (PMC13283981; doi:10.3389/fped.2026.1821139)
Supplement: Supplementary file 2 [file Table2.docx]

**Supplementary Table S2. Stage-stratified clinical characteristics and exploratory hepcidin signal analysis.**

| Variable | non-ROP, *n* = 24 | ROP 1, *n* = 5 | ROP2, *n* = 4 | ROP 3, *n* = 2 | Overall *P* value |
| --- | --- | --- | --- | --- | --- |
| Gestational age,  weeks | 31.10 ± 2.31 | 29.14 ± 1.93 | 28.93 ± 3.79 | 26.14 ± 1.21 | 0.023 |
| Birth weight, g | 1527.08 ± 435.29 | 1228.00 ± 311.24 | 1252.50 ± 513.90 | 852.50 ± 201.53 | 0.102 |
| Raw ELISA-  derived hepcidin  signal, pg/mL | 50.21 ± 11.79 | 37.49 ± 11.12 | 46.63 ± 9.85 | 42.65 ± 9.31 | 0.159 |
| Hepcidin below  LLOQ | 21/24 (87.5%) | 5/5 (100.0%) | 4/4 (100.0%) | 2/2 (100.0%) | — |
| Hepcidin below  LOD | 3/24 (12.5%) | 2/5 (40.0%) | 1/4 (25.0%) | 1/2 (50.0%) | — |

Table note: Data are presented as mean ± SD or n/N (%). Stage-stratified comparisons were performed using one-way ANOVA for continuous variables. LLOQ was defined as 62.5 pg/mL, corresponding to the lower limit of the reported quantitative range of the ELISA assay. LOD was defined as 37.5 pg/mL, corresponding to the reported sensitivity threshold of the assay. Raw ELISA-derived hepcidin signals are shown for exploratory description only because most values were below the LLOQ. The stage-stratified analysis should be interpreted cautiously because the ROP stage subgroups were small, especially stage 3 ROP.
