# Supplementary material for: Serum hepcidin is associated with retinopathy of prematurity and modulates oxidative stress and angiogenic responses in retinal microvascular endothelial cells
Source: Front Pediatr. 2026 Jun 8;14:1821139. doi: 10.3389/fped.2026.1821139 (PMC13283981; doi:10.3389/fped.2026.1821139)
Supplement: Supplementary file 3 [file Table3.docx]

**Supplementary Table S3. Prematurity-related comorbidities and potential confounders in infants with and without ROP.**

| **Clinical factor** | **non-ROP, *n* = 24** | **ROP, *n* = 11** |
| --- | --- | --- |
| Bronchopulmonary dysplasia | 10/24 (41.7%) | 8/11 (72.7%) |
| Intracranial or intraventricular hemorrhage | 10/24 (41.7%) | 7/11 (63.6%) |
| Anemia | 17/24 (70.8%) | 8/11 (72.7%) |
| Iron supplementation | 10/24 (41.7%) | 7/11 (63.6%) |
| Infection or inflammatory conditions | 10/24 (41.7%) | 9/11 (81.8%) |
| NEC or severe gastrointestinal disease | 8/24 (33.3%) | 7/11 (63.6%) |
| Patent ductus arteriosus | 6/24 (25.0%) | 2/11 (18.2%) |
| Pulmonary hypertension | 2/24 (8.3%) | 1/11 (9.1%) |
| Hemangioma | 0/24 (0.0%) | 2/11 (18.2%) |
| Acute kidney injury or renal failure | 1/24 (4.2%) | 2/11 (18.2%) |
| Cholestasis or hepatitis | 4/24 (16.7%) | 2/11 (18.2%) |

**Table note:** Data are presented as n/N (%). Comorbidities were extracted from clinical diagnoses and medical records. Categories were not mutually exclusive. No clearly documented primary systemic vascular disease, such as systemic vasculitis or hereditary systemic vascular malformation, was identified in the ROP cohort. Infection or inflammatory conditions included clinically documented infection, pneumonia, sepsis, meningitis, abscess, or peritonitis. NEC or severe gastrointestinal disease included necrotizing enterocolitis, intestinal obstruction, gastrointestinal perforation, peritonitis, major congenital gastrointestinal disease, or postoperative gastrointestinal disease. These variables were summarized as prematurity-related comorbidities and potential clinical confounders relevant to the interpretation of serum hepcidin findings.
