# Supplementary figures and images for: Serum hepcidin is associated with retinopathy of prematurity and modulates oxidative stress and angiogenic responses in retinal microvascular endothelial cells
Source: Front Pediatr. 2026 Jun 8;14:1821139. doi: 10.3389/fped.2026.1821139 (PMC13283981; doi:10.3389/fped.2026.1821139)

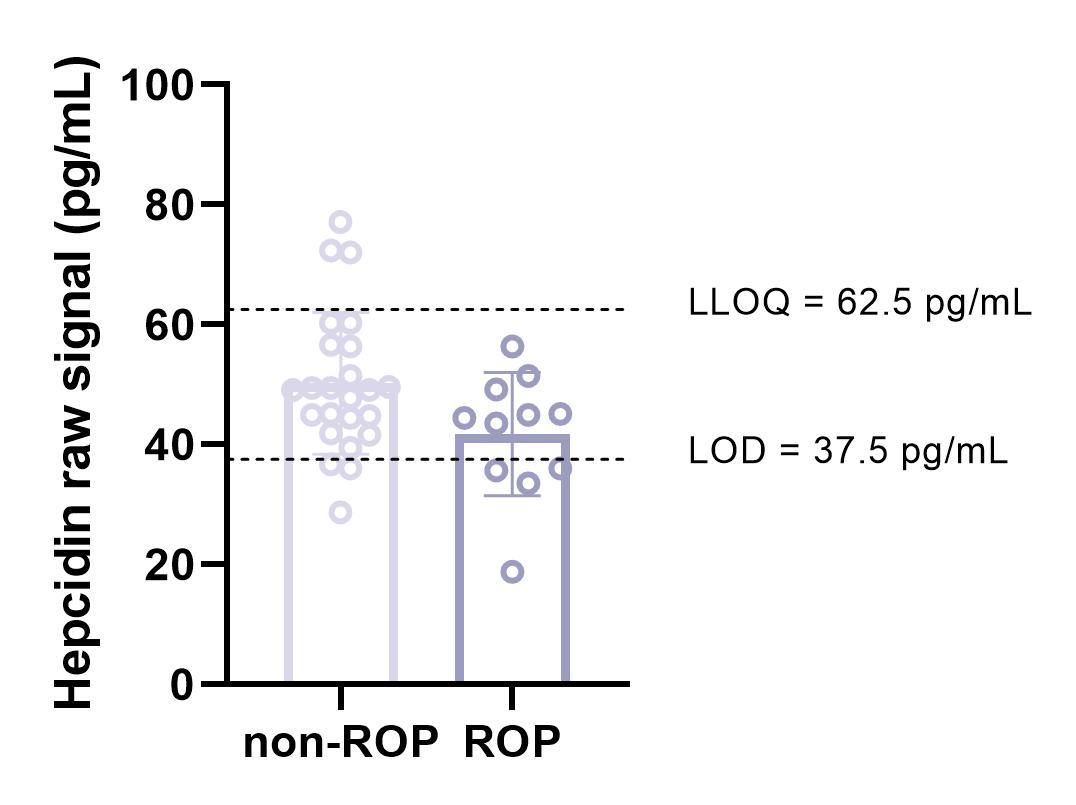

Supplement: Supplementary file 4 [file Image1.jpeg]
